# Protective mechanism of FoxO1 against early brain injury after subarachnoid hemorrhage by regulating autophagy

**DOI:** 10.1002/brb3.2376

**Published:** 2021-10-17

**Authors:** Haitao Hao, Yahui Bai, Yu Liu, Junxin Liang, Shichao Guo

**Affiliations:** ^1^ Department of Neurosurgery The First Affiliated Hospital of Zhengzhou University Zhengzhou Henan P. R. China; ^2^ Henan Key Laboratory of Neurorestoratology The First Affiliated Hospital of Xinxiang Medical University Weihui Henan P. R. China

**Keywords:** after subarachnoid hemorrhage, autophagy, early brain injury, FoxO1

## Abstract

**Introduction:**

Early brain injury (EBI) plays a key role in the devastating outcomes after subarachnoid hemorrhage (SAH). Autophagy and apoptosis may share a common molecular inducer that regulates the process of cell death. FoxO1, as a key regulator of neuronal autophagy which is involved in apoptosis, has not been reported in SAH rats. This work was to investigate the protective and anti‐inflammatory effects of FoxO1 on EBI after SAH by regulating autophagy.

**Methods:**

In this study, we constructed the SAH model. In experiment I, low dose (50 μl of 1 × 10^8^ IU/ml) or high dose (50 μl of 1 × 10^10^ IU/ml) of FoxO1 gene overexpressed adenovirus vector was injected into the lateral ventricle of rats before SAH. In experiment II, we reported the effect of FoxO1 overexpress on nerve function recovery, oedema, BBB leakage, neuronal death in rats after SAH through autophagy regulation. Post‐SAH evaluation included neurological function score, brain water content, evans blue exosmosis, pathological changes, inflammatory response and apoptosis.

**Results:**

The experiment I showed that either low or high dose of ad‐FoxO1 could significantly improve nerve function, reduce cerebral water content and reduce blood‐brain barrier (BBB) destruction in rats, indicating that ad‐FoxO1 had a protective effect on brain injury in rats EBI after SAH. In addition, ad‐FoxO1 promoted autophagy in rat hippocampal tissue, as evidenced by accumulation of LC3II/I and Beclin‐1 and degradation of p62. Furthermore, ad‐FoxO1 inhibited the inflammatory response and apoptosis of rat hippocampal neurons after SAH. The experiment II showed that both ad‐FoxO1 and rapamycin attenuated the injury of nerve function in rats after SAH, and this synergistic effect further reduced cerebral edema and evansblue extravasation, decreased hippocampus neuronal cell apoptosis, and declined inflammatory response. However, this was contrary to the results of chloroquine. These findings suggested that FoxO1 regulated the neural function of EBI after SAH through the autophagy pathway.

**Conclusions:**

The findings in this study was beneficial for identifying the novel therapeutic target for the treatment of SAH.

## Graphical abstract

Our study extends the current understanding of autophagy in SAH‐induced early brain injury. We demonstrate that FoxO1 plays a protective role in early brain injury after SAH by involving in enhancing autophagy flux. This neuroprotective effect may be associated with autophagy mediated anti‐apoptotic mechanisms, which may provide new insights for SAH therapy.

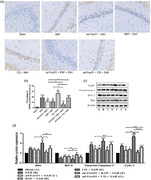

## 1 INTRODUCTION

Subarachnoid hemorrhage (SAH) is a devastating subtype of hemorrhagic stroke with high mortality and disability (Gathier et al., 2015). Some studies have pointed out that cerebral hemorrhage causes brain edema, produces inflammatory damage, destroys the blood–brain barrier (BBB), increases intracranial pressure, and eventually leads to nerve cell necrosis (Yang J. & Wang, 2021; Zhang L. et al., 2021). Accumulated evidence has shown that early brain injury (EBI) induced by microglia activation releases a large number of inflammatory cytokines, which can be used as a key indicator of the inflammatory response in the brain (Li P., Li X. et al., 2020).

Autophagy is a highly regulated way of intracellular component degradation, which plays an important role in maintaining normal cell function, growth, and development (Yang Z. & Klionsky, 2010). A wide range of intracellular and extracellular stimuli, including amino acid deficiency and microbial invasion, can lead to an autophagic response. Autophagy is associated with a variety of pathological processes, including cancer and neurologic decline (Kim, 2021; Zhou et al., 2021). Studies have shown that autophagy is a normal physiological process in neurons, which is constitutively active at the basal level. In animal models, the blocking of basal autophagy leads to the death of neurons (Nikoletopoulou et al., 2015). Importantly, a large amount of evidence indicates that there is an interaction between autophagy and apoptosis (Guo D. et al., 2018; Li T. et al., 2015; Loos et al., 2011), which play important roles in maintaining developmental processes, tissue homeostasis, and the pathogenesis of many diseases. So far, there is increasing evidence that the major molecular components of autophagy and apoptosis signaling pathways are involved in complex crosstalk, and they are usually induced by similar stimuli. For example, experiments have shown that both apoptosis and autophagy are activated in response to metabolic stress (Shliapina et al., 2021) or exposure to reactive oxygen species (Poillet‐Perez et al., 2015). Besides, autophagy pathway in brain is activated after the experimental subarachnoid hemorhage (SAH), which plays an important role in its EBI (Li X. et al., 2018). Autophagy precedes apoptosis, which has a protective effect in the early stages of programmed cell death (Jellinger & Stadelmann, 2000). However, it can also promote apoptosis in some cases (Zhu et al., 2021). Recently, it has been reported that autophagy begins to increase at 6 h after SAH and continues throughout the early stage of brain injury (Sun et al., 2019). However, the role of autophagy in the pathogenesis of EBI following SAH is not fully understood.

Forkhead box protein O1 (FoxO1) is an important member of the FoxO transcription factor family, which regulates a variety of molecular signals in tissues (Schiattarella & Altamirano et al., 2021). These transcription factors are highly expressed in the brain and are the primary mediators for regulating autophagy. Previous studies have shown that FoxO1 decreases macrophage inflammation (Tsuchiya et al., 2011). Besides, endogenous FoxO1 is a key pathway molecule for apoptosis and autophagy due to oxidative stress (Zhao Y. et al., 2010), and also a key regulator of autophagy in neurons (Vidal et al., 2012). Recently, increasing evidence demonstrates that FoxO1 has a unique function in inducing autophagy in various diseases (Hariharan et al., 2010; He et al., 2017).

However, it remains largely uncertain the exact mechanisms responsible for the protective and anti‐inflammatory effects of FoxO1 on EBI after SAH by regulating autophagy. In this study, we investigated the effect of FoxO1 overexpress on nerve function recovery, oedema, BBB leakage, neuronal apoptosis, and autophagy activation in rats after SAH during EBI. This study may provide a theoretical basis for the development of novel therapeutic targets for SAH treatment.

## 2 MATERIALS AND METHODS

### 2.1 Animal study

Male Sprague‐Dawley rats (350–450 g) were purchased from the animal center of the Chinese Academy of Sciences (Beijing, China). Rats were maintained under standard laboratory condition, with free access to food and water, and housed prior to experiments in an animal room under standard conditions (23 ± 2°C; 55 ± 5% humidity; 12 h light/dark cycle). The rats were free of all viral, bacterial, and parasitic pathogens. Experimental animals were not used for breeding purposes. All animal experiments were approved by the Ethics Committee of the First Affiliated Hospital of Xinxiang Medical College.

### 2.2 Rat SAH model

The rat SAH model was designed by injecting blood into the prechiasmatic cistern as described previously (Wang Z. et al., 2012). Briefly, rats were anesthetized by an intraperitoneal injection of 3% pentobarbital sodium (35 mg/kg). Subsequently, the prone position was fixed to the stereoscopic locator, the forehead was routinely disinfected, the surface skin was cut open, and the muscle and periosteum were dully separated. Under the principle of aseptic operation, 0.3 ml of fresh autogenous non‐heparinized femoral artery artery blood was slowly injected into the crossed anterior cistern for 20 s in the SAH group with an injection pump. The sham group underwent the same operation as the SAH group, and only the injection of autogenous non‐heparinized femoral artery blood was replaced with an equivalent amount of 37℃ normal saline.

### 2.3 Experimental groups and drug administration

Two separate experiments were conducted in this study. Experiment I was designed to assess the effect of FoxO1 on EBI after SAH induction and determine the optimal dose of FoxO1 overexpressed adenovirus for subsequent experiments. The rats were randomly divided into 4 groups: sham group, SAH group, FoxO1 overexpressed adenovirus low‐dose (50 μl of 1 × 10^8^ IU/ml) +SAH group, and FoxO1 overexpressed adenovirus high‐dose (50 μl of 1 × 10^10^ IU/ml) +SAH group, among which the corresponding doses of FoxO1 overexpressed adenovirus were injected into the lateral ventricle of rats for 1 week before molding. In addition, the sham group was injected with the same amount of adenovirus negative control (ad‐NC) at corresponding sites.

Experiment Ⅱ was designed to investigate the effect of FoxO1 overexpress on nerve function recovery, oedema, BBB leakage, neuronal death in rats after SAH through autophagy regulation. The rats were randomly divided into 7 groups: sham group, SAH group, ad‐FoxO1+SAH group, RAP+SAH group, CQ+SAH group, ad‐FoxO1+RAP+SAH group, and ad‐FoxO1+CQ+SAH group, among which the lateral ventricle dose of FoxO1 overexpressed adenovirus was 50 μl of 1 × 10^10^ IU/ml/d, accompanied by the same amount of ad‐NC adenovirus injection in sham group, and the intraperitoneal dose of autophagy inducer (rapamycin, RAP) or autophagy inhibitor (chloroquine, CQ) was 20 mg/kg/d, respectively. All drugs were administered continuously for 1 week before molding.

All rats were sacrificed after 24 h of modeling. Hippocampal tissue was immediately removed, rinsed with physiological saline solution, and then stored at −80°C prior to further analysis.

### 2.4 Neurobehavioral function assessment

The modified Garcia scoring system was used to blind measure the neurobehavioral function in this study, which was consistent with previous studies (Marbacher, 2016). In short, the evaluation included six test items: spontaneous activity (score, 0–3), climbing (score, 1–3), forelimb stretching (score, 0–3), spontaneous movements of all limbs (score, 0–3), body proprioception (score, 1–3), and tactile response to vibration (score, 1–3). The total neurological score was calculated as the sum of all sectors, and the possible scores range from 3 to 18, depending on the severity of the neurological defect. The lower the score, the more severe the neurological impairment caused by SAH.

### 2.5 Brain edema measurement

The brain moisture content was determined by the standard wet‐dry method. Briefly, after the rats were sacrificed, the brains were immediately separated and weighed for weight determination. The brain was then dried at 105°C for 72 h, which is called dry weight. Finally, the percentage of brain water content was calculated as follows: (Wet weight − Dry weight)/Wet weight × 100%.

### 2.6 BBB permeability

BBB permeability was examined by the extravasation of Evans blue dye at 24 h after SAH. The anesthetized rats were intravenously injected with 4% Evans blue (EB) solution (2.5 ml/kg) via the tail vein an hour before the sacrifice. After 1 h of systemic circulation, thoracotomy was performed and the heart was exposed after the capsule was removed. With the infusion needle pierced the left ventricle, the ascending aorta was inserted and the heart was fixed with bent forceps. Then, 0.1 M PBS was infused into the left ventricle, keeping perfusion pressure was controlled at 100 mmHg. When the infusion becomes clear, the brain is severed to collect specimens. After weighing, the tissues of the left and right hemispheres were immersed in formamide solution in a ratio of 10 ml/kg, and then homogenized. The homogenate was placed in a constant temperature water bath at 60℃ and incubated for 24 h. After EB leaching, the absorbance (A_615_) value of the leaching solution was determined at the wavelength of 615 nm of the enzyme marker (Thermo Fisher Scientific, MA, USA). The amount of Evans blue in the brain tissue was calculated according to the standard curve.

### 2.7 Western blot assay

Western blot analysis was performed to detect protein expression in hippocampus tissues. Protein extraction kit (Pierce, Thermo Fisher, Ltd.) was used to extract protein. The concentration of the sample was determined according to the instructions of KCTMBCA protein quantitative kit. According to the total protein concentration measured, equal amounts of proteins (30 μg) with different molecular weights in the lysate were then separated adopting 10% SDS‐PAGE and transferred onto PVDF membrane (Thermo Fisher Scientific, Inc.). Subsequently, the membrane was blocked with 5% skim milk and probed with primary antibodies: rabbit anti‐Bcl‐2 (ab196495; 1:500), rabbit anti‐Bax (ab53154; 1:500), rabbit anti‐Cleaved‐Caspase3 (ab2302; 1:1,000), rabbit anti‐Cytochrome C (ab133504; 1:5,000), rabbit anti‐Beclin‐1 (ab207612; 1:2,000), rabbit anti‐p62 (ab109012; 1:10,000), rabbit anti‐LC3B (ab51520; 1:3,000), and rabbit anti‐β‐actin (ab8227; 1:1,000). The membrane was incubated with horseradish peroxidase‐labeled goat anti‐rabbit immunoglobulin G (IgG;1:2000, ab205718) at room temperature for 1 h. After X film exposure and development, Bio‐Rad automatic gel imaging system was used for imaging preservation. Using ImageJ image analysis software to analyze gray scale, the gray value of the target protein was divided by the internal reference gray value to correct the error. The relative content of target protein was analyzed statistically. β‐actin was served as inner loading control.

### 2.8 Histological examination

The tissue samples were carefully removed and fixed in 4% (v/v) paraformaldehyde at room temperature for 48 h. The tissue was embedded in paraffin and cut into 4‐μm sections. The sections were heated at 60°C for 1 h and then dewaxed with xylene. Following hydration, the sections were stained with 0.5% H&E at room temperature for 5 min, dehydrated with gradient ethanol, cleared with xylene, and mounted with neutral gum. Optical microscopy (BA400 Digital, McAldy industrial group co. LTD) was used to examine pathological changes of the brain tissue (magnification, ×100 or ×400). The pathological changes of hippocampal neurons were observed under the light microscope.

### 2.9 Toluidine blue staining

Toluidine blue staining was carried out as before. Simply, 2–3 mm of hippocampal nerves were obtained from the proximal end of the impaired hippocampal nerve. The nerves were immobilized in cold glutaraldehyde (3% w/v) and infiltrated with a graded araldite epoxy propane mixture. After dehydration with ethanol and 1% osmium tetroxide, they were embedded in epon812 (Shell Chemical, CA, USA). Subsequently, the ganglion segments were immobilized and then stained with 1% toluidine blue. Finally, the results were examined by light microscope (Olympus BX60, Tokyo, Japan). The DP12 camera (Olympus) with 100 × oil‐immersed objective lens was used for image acquisition. Images Pro‐Plus 3.0 (MediaCybernetics, Bethesda, MD, USA) was used for image processing. The number of myelin axons in the cavernous nerve was analyzed.

### 2.10 TUNEL staining

The neuronal apoptosis in hippocampus was detected by TUNEL staining. According to the TUNEL kit manual operation, the paraffin sections of the dewaxed hydrated brain were sealed by 3% hydrogen peroxide solution at room temperature for 15 min, on which 0.1% citric acid solution containing 0.1% Triton X‐100 was dropped, and then the samples were placed on ice for reaction for 3 min. Subsequently, 50 μl TUNEL reaction mixture was added to each sample and incubated at 37℃ in a wet box for 1 h. Another 100 ug/L DAPI was added for 8 min for nuclear staining, afterward the visual field radiography was selected under a fluorescence microscope (OLYMPUS, BX51TF). The apoptotic cell count was performed by a pathologist who did not understand the grouping. Apoptotic index was determined by the ratio of the number of TUNEL positive neurons to the total number of neurons.

### 2.11 Determination of inflammatory factors IL‐6, TNF‐α, COX2, and IL‐1β levels in hippocampus

Levels of inflammatory factors IL‐6 (ab100712), TNF‐α (ab208348), COX2 (ab210574), and IL‐1β (ab197742) in hippocampus were determined by ELISA Kits all from Abcam (Cambridge, MA, UK) according to the manufacturer's instructions. A blank well and a sample well were set up respectively. The optical density (OD) of each well was measured at 450 nm, and the concentration of inflammatory cytokines was quantified in accordance with the standard curve.

### 2.12 Statistical analysis

Data were expressed as mean ± standard deviation. SPSS 17.0 (SPSS, Inc., Chicago, IL, USA) was used for statistical evaluation. Student's *t*‐test or one‐way analysis of variance was used to estimate the statistical differences among groups. *p* < .05 was considered to indicate a statistically significant difference.

## 3 RESULTS

### 3.1 Ad‐FoxO1 alleviated brain edema and improved neurological function during EBI following SAH

**FIGURE 1:**
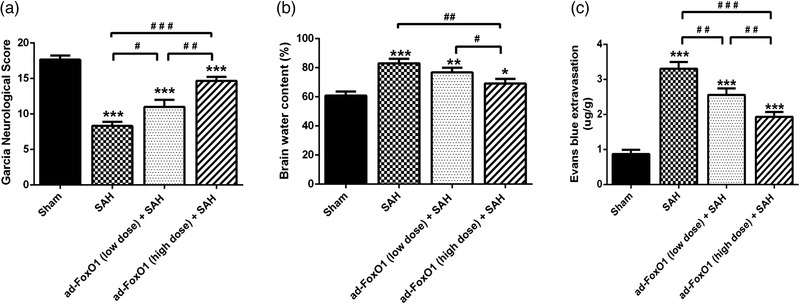
Both low and high doses of ad‐FoxO1 can alleviate brain edema and improve neurological function after SAH. (a) The neurological function was evaluated by the Garcia neurological score after SAH. (b) Brain water content in each group. (c) Content of Evans blue extravasation in each group. *n* = 6 in each group, *p < .05, **p < .01, ***p < .001, vs. sham group. ^#^ *p* < .05, ^##^ *p* < .01, ^###^ *p* < .001

To investigate the effect of FoxO1 on the SAH rats models, high dose or low dose ad‐FoxO1 was administered continuously for 1 week before SAH induction respectively, which would inform the optimum medicinal dose for ad‐FoxO1 treatment in the subsequent studies. Twenty‐four hours following SAH induction, compared with the sham group, the Garcia neurological score of SAH groups all showed a significant decrease in different degrees. In addition, compared with the SAH group, there was a significantly gradient increasing trend of Garcia neurological score in the ad‐foxo1 (low dose)+SAH group and ad‐foxo1 (high dose)+SAH group, respectively. Clearly, the high‐dose ad‐FoxO1 (high dose) group showed the best improvement in Garcia neurological score compared to the SAH group (Figure 1a).

The change of SAH is initially a physiological regulatory mechanism to reduce intracranial hemorrhage, which can easily lead to brain edema and eventually nerve cell damage (Guo S. et al., 2020; Zhang X. S. et al., 2016). Therefore, determination of brain water content in SAH rat could investigate whether ad‐FoxO1 was effectively improved neurological function. As shown in Figure 1b, at 24 h post‐SAH, the brain water content was markedly increased in SAH groups compared to Sham group, while compared with ad‐FoxO1 (high dose)+SAH group, the brain water content of ad‐FoxO1 (low dose)+SAH group and SAH group increased significantly (Figure 1b).

The destruction of the BBB is a consequence of apoptosis and necrosis (Wei et al., 2020). Here, we used Evans blue method to evaluate the degree of destruction of the BBB. As shown in Figure 1c, the content of Evans blue extravasation in SAH groups was significantly higher than that in Sham group. Compared with SAH group, there was a significant decrease after administration of different doses of ad‐FoxO1 by instillation. Additionally, the greatest reduction of Evans blue content was seen at the high doses (Figure 1c).

### 3.2 Ad‐FoxO1 improved pathomorphology during EBI following SAH

**FIGURE 2:**
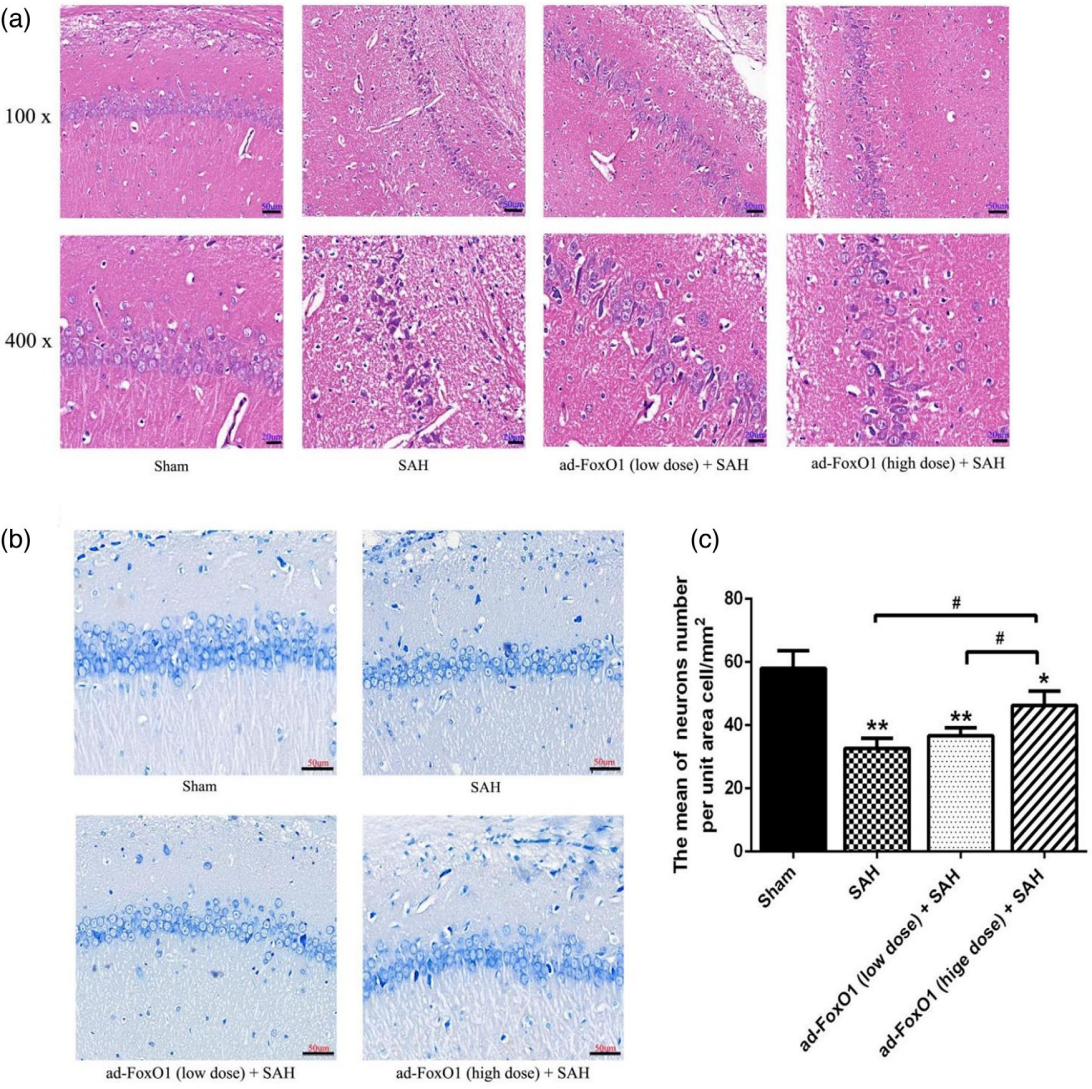
Both low and high doses of ad‐FoxO1 can improve pathomorphology and protect neurons from injury. (a) HE staining showed the pathomorphology of hippocampus following ad‐FoxO1 treatment after SAH. (b) Toluidine blue staining showed the qualitative histological changes of hippocampus following ad‐FoxO1 treatment after SAH (400×). (c) The neurons number per unit area cell/mm^2^ showed a significant increase of ad‐FoxO1 treated group relative to SAH group in hippocampus. *n* = 6 in each group, ***p < .001, vs. sham group. ^##^ *p* < .01, ^###^ *p* < .001

HE staining revealed the pathological changes of hippocampal neurons in the rats. As regards the Sham group, there was no obvious abnormality in the brain tissue of the Sham group, among which, the pyramidal cell layer in the CA1 region of the hippocampus was densely packed with cells, the morphology of neurons was normal, the color of cell nuclei was lightly stained, and no obvious degeneration or necrosis was observed (Figure 2a). Compared with the Sham group, the other three groups of SAH model rats all had different degrees of lesions, among which the SAH group had a large number of necrotic or decreased vertebral cells, with smaller volume, deeper staining, and cytosolic coagulation after cell necrosis. Ad‐FoxO1 (low dose)+SAH group and ad‐FoxO1 (high dose)+SAH group had different degrees of alleviate symptoms, among which, both groups were accompanied by smaller necrotic cells, whose nuclei were solidified and stained deeply (Figure 2a). The vertebral cells in the low dose group were still partially necrotic and disorganized. Although the number of vertebral somatic cells in the high‐dose group was partially reduced, it improved the symptoms of SAH (Figure 2a).

The number of neurons in the hippocampus was determined by toluidine blue staining. Toluidine blue staining results showed that there was no obvious neuronal injury in the sham group. However, when compared with the sham group, rats in the SAH group were exhibited a significant increase in the neuronal apoptosis index and a decrease in the number of normal neurons. Ad‐FoxO1 treatment, however, significantly inhibited the number of apoptotic neurons when compared with the SAH group. Additionally, rats treated with the high‐dose ad‐FoxO1 appeared to achieve less neuronal apoptosis compared to the low‐dose ad‐FoxO1 treatment group (Figure 2b,c). This suggested that ad‐FoxO1 protected neurons from injury.

### 3.3 Ad‐FoxO1 reduced positive neurons apoptosis during EBI following SAH

**FIGURE 3:**
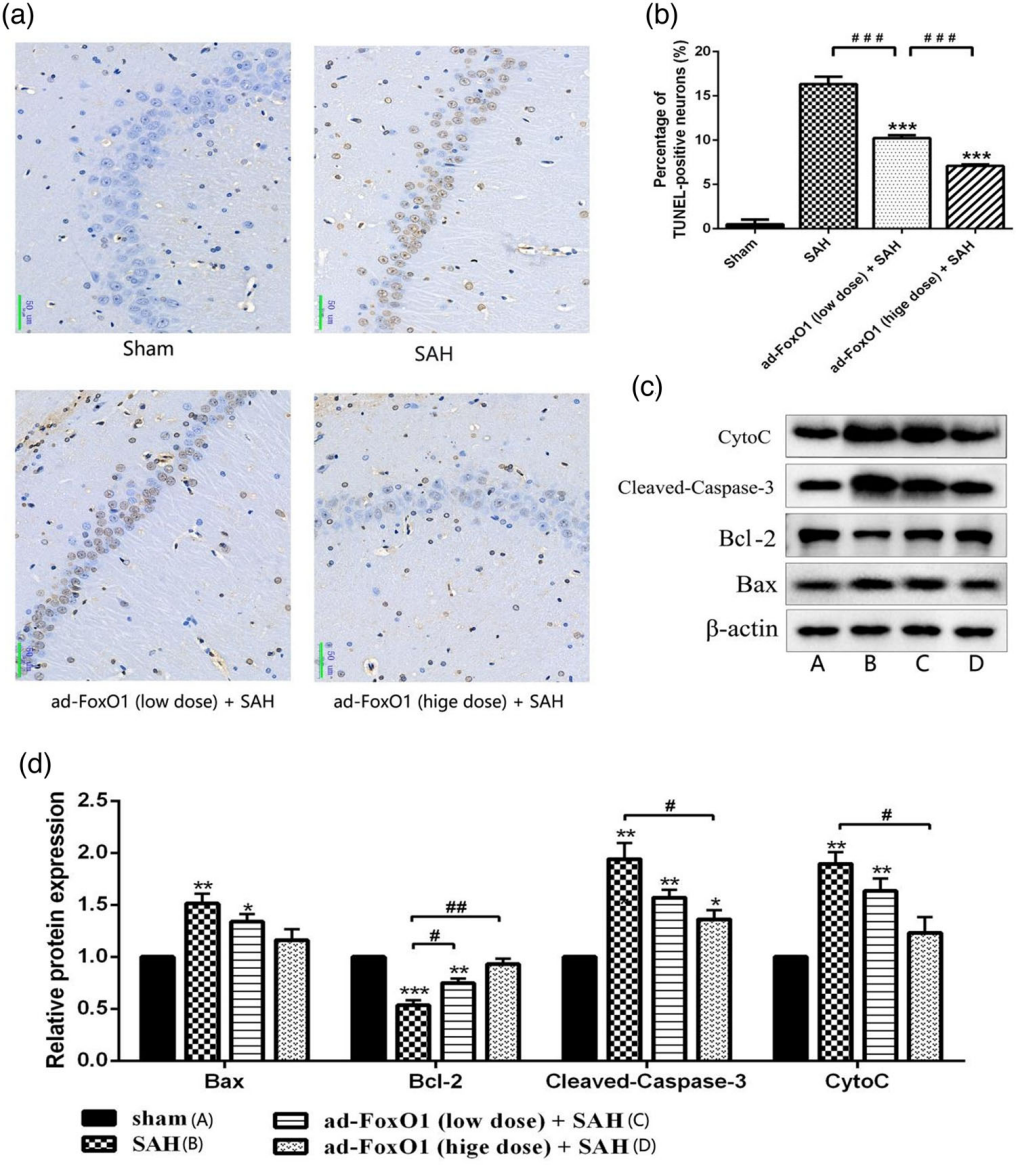
Effects of ad‐FoxO1 on neural apoptosis in SAH rats. (a) Representative images of hippocampus following TUNEL staining, TUNEL‐positive cells were stained brown (400×). (b) Represents the change of neuronal apoptosis rate in each group. (c) Apoptosis‐related proteins were examined by western blotting assay. (d) The relative intensity of apoptosis‐related proteins was shown as a bar graph. *n* = 6 in each group, *p < .05, **p < .01, ***p < .001, vs. sham group. ^#^ *p* < .05, ^##^ *p* < .01

Many experiments have shown that apoptosis may play an important role in EBI (Liu L. et al., 2021). During cerebral ischemia, the concentration of free radicals exceeds the local antioxidant capacity, causing brain damage and vascular membrane destruction, leading to cell apoptosis (Li X., Liu W. et al., 2020). As shown in Figure 3a,b, TUNEL‐positive cells were hardly detected in sham group, but many TUNEL‐positive cells were detected in the hippocampal neuronal apoptosis of SAH rats. However, low dose and high dose ad‐FoxO1 both significantly reduced the number of TUNEL‐positive neural cells, with the high‐dose group maximizing the inhibition of apoptosis as the optimal dose. Furthermore, compared with SAH group, western blot results revealed that high dose ad‐FoxO1 significantly decreased the expression of Cleaved‐caspase3 and CytoC proteins. Besides, the low dose and high ad‐FoxO1 both significantly increased the expression of Bcl‐2. In addition, the expression of Bax showed no significant difference, although there was a downregulation trend (Figure 3c,d). In summary, these data suggested that ad‐FoxO1 has a neuroprotective effect against apoptosis on SAH.

### 3.4 Ad‐FoxO1 attenuated the contents of inflammation‐related cytokines and promoted autophagy during EBI following SAH

**FIGURE 4:**
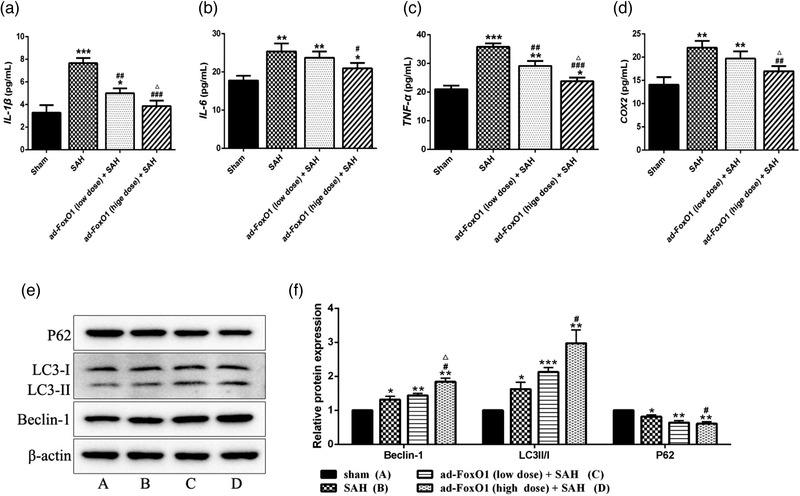
Effects of ad‐FoxO1 on inflammatory factor and autophagy‐related factors in SAH rats. (a) The content of IL‐1β in each group. (b) The content of IL‐6 in each group. (c) The content of TNF‐α in each group. (d) The content of COX‐2 in each group. (e) Representative western blot bands for autophagy‐related proteins 24 h after SAH. (f) Relative protein expression of autophagy‐related proteins in hippocampus in each group. The results were presented as the mean ± standard deviation. *n* = 6 in each group, *p < .05, **p < .01, ***p < .001 vs. sham group. ^#^ *p* < .05, ^##^ *p* < .01, ^###^ *p* < .001 vs. SAH group. ^△^ *p* < .05 vs. ad‐FoxO1 (low dose) group

The effect of ad‐FoxO1 on the release of inflammatory cytokines in SAH rats was determined by ELISA assay. The results revealed that the levels of IL‐1β, IL‐6, TNF‐α, and COX2 were significantly decreased by ad‐FoxO1 treatment compared with those in SAH group. Besides, high‐dose of ad‐FoxO1 has no effect on the inflammatory response in normal rats (Figure 4a–d). These results indicated that ad‐FoxO1 can improve inflammation of brain tissues in SAH rats.

At present, autophagy induction is considered to be an important way to promote the removal of abnormal protein accumulation by neurons, along with the maintenance of cell survival. However, it is not clear whether ad‐FoxO1 is involved in autophagy to improve neural function in SAH rats. Conversion of LC3B‐I to LC3B‐II is an indicator of autophagosome formation. p62 is an adapter protein that links aggregated proteins sequestered in autophagosomes and is degraded in autolysosomes. Therefore, increase level of p62 is usually considered an indicator of impaired autophagic flux. As is known from Figure 4e,f, the protein expressions of Beclin‐1 and LC3II/I ratio increased significantly in SAH group compared with the sham group, while the protein expression of p62 decreased remarkably, suggesting that the autophagy flux in the SAH rats may be activated. In addition, the protein expressions of Beclin‐1 and LC3II/I increased significantly and p62 decreased significantly in the low‐dose or high‐dose ad‐FoxO1 group compared with the SAH group. Of these, the high‐dose group showed an optimal effect of promoting autophagy. This indicated that the autophagy pathway was further promoted under the treatment of ad‐FoxO1 in SAH rats.

### 3.5 Ad‐FoxO1 involved in autophagy regulation to improve cerebral edema and nerve function during EBI following SAH

**FIGURE 5:**
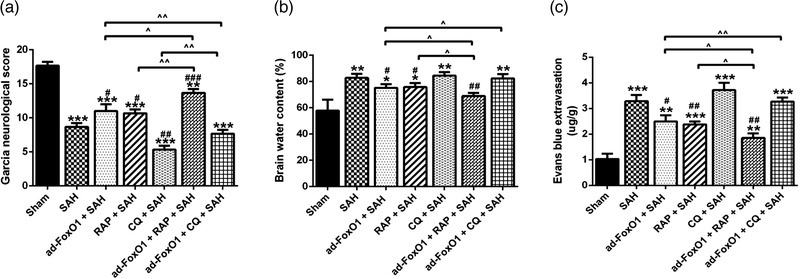
Ad‐FoxO1 alleviated brain edema and improved neurological function by regulating autophagy 24 h after SAH. (a) The neurological function was evaluated by the Garcia neurological score after SAH. (b) Brain water content in each group. (c) Content of Evans blue extravasation in each group. *n* = 6 in each group, **p < .01, ***p < .001, vs. sham group. ^#^ *p* < .05, ^##^ *p* < .01, ^###^ *p* < .001, vs. SAH group. ˆ*p* < .05, ˆˆ*p* < .01

The above confirmation indicates that ad‐FoxO1 has a protective effect on nerve within 24 h after SAH, so we speculate that the increase of FoxO1 may have a self‐protective effect on the body. In addition, studies have shown that FoxO1, as an autophagy regulator, can be used as protective autophagy to protect the body before 48 h after SAH. Therefore, we investigated whether ad‐FoxO1 had an effect on neuronal function through involving in autophagy. Here, we reported the regulation mechanism of the interaction between ad‐FoxO1 and RAP or CQ on EBI 24 h after SAH. Results showed that the hippocampus Garcia neurological score of rats in sham group was significantly higher than that of any other group, which consistent with our expectations. The hippocampus Garcia neurological score of SAH group was significantly lower than in RAP+SAH group, and significantly higher than that in CQ+SAH group, which showed that RAP had neuroprotective effects on rats 24 h after SAH, while CQ had the opposite effect. Consistented with the above findings, SAH rats receiving ad‐FoxO1 showed a significant increase of Garcia neurological score. It was interesting to note that, compared with the ad‐FoxO1+SAH group, the Garcia neurological score significantly increased in ad‐FoxO1+RAP+SAH group, while the Garcia neurological score significantly decreased in ad‐FoxO1+CQ+SAH group (Figure 5a).

As shown in Figure 5b, compared with the sham group, there was a significant increase of brain water content in all the other groups (except ad‐FOXO1+RAP+SAH group). What's more, compared with the SAH group, there was a trend of significant decrease of brain water content in the ad‐FoxO1+SAH group, RAP+SAH group, and ad‐FoxO1+RAP+SAH group. Besides, compared with ad‐FoxO1+SAH group, the brain water content in ad‐FoxO1+RAP+SAH group significantly reduced, while the brain water content in the ad‐FoxO1+CQ+SAH group significantly increased. Furthermore, brain water content in RAP+SAH group significantly higher than ad‐FoxO1+RAP+SAH group (Figure 5b).

Evans blue extravasation was significantly higher in SAH group than in sham group. SAH rats receiving either ad‐FoxO1 or RAP treatment showed a significant inhibition of Evans blue extravasation, while SAH rats receiving CQ showed a significant increase of Evans blue extravasation. Furthermore, compared with ad‐FoxO1+SAH group, ad‐FoxO1+RAP+SAH group demonstrated a significant decrease of Evans blue extravasation, while ad‐FoxO1+CQ+SAH group demonstrated a significant increase of Evans blue extravasation. What's more, the ad‐FoxO1+RAP+SAH group showed a further reduction of Evans blue extravasation compared to the RAP+SAH group. These results suggested that ad‐FoxO1 regulating autophagy had an effect on BBB function of rats during EBI during SAH (Figure 5c). These results revealed that ad‐FoxO1 may be involved in autophagy regulation to improve nerve function of rats after SAH.

### 3.6 Ad‐FoxO1 involved in autophagy regulation to improve pathomorphology and inhibited neuronal apoptosis during EBI following SAH

**FIGURE 6:**
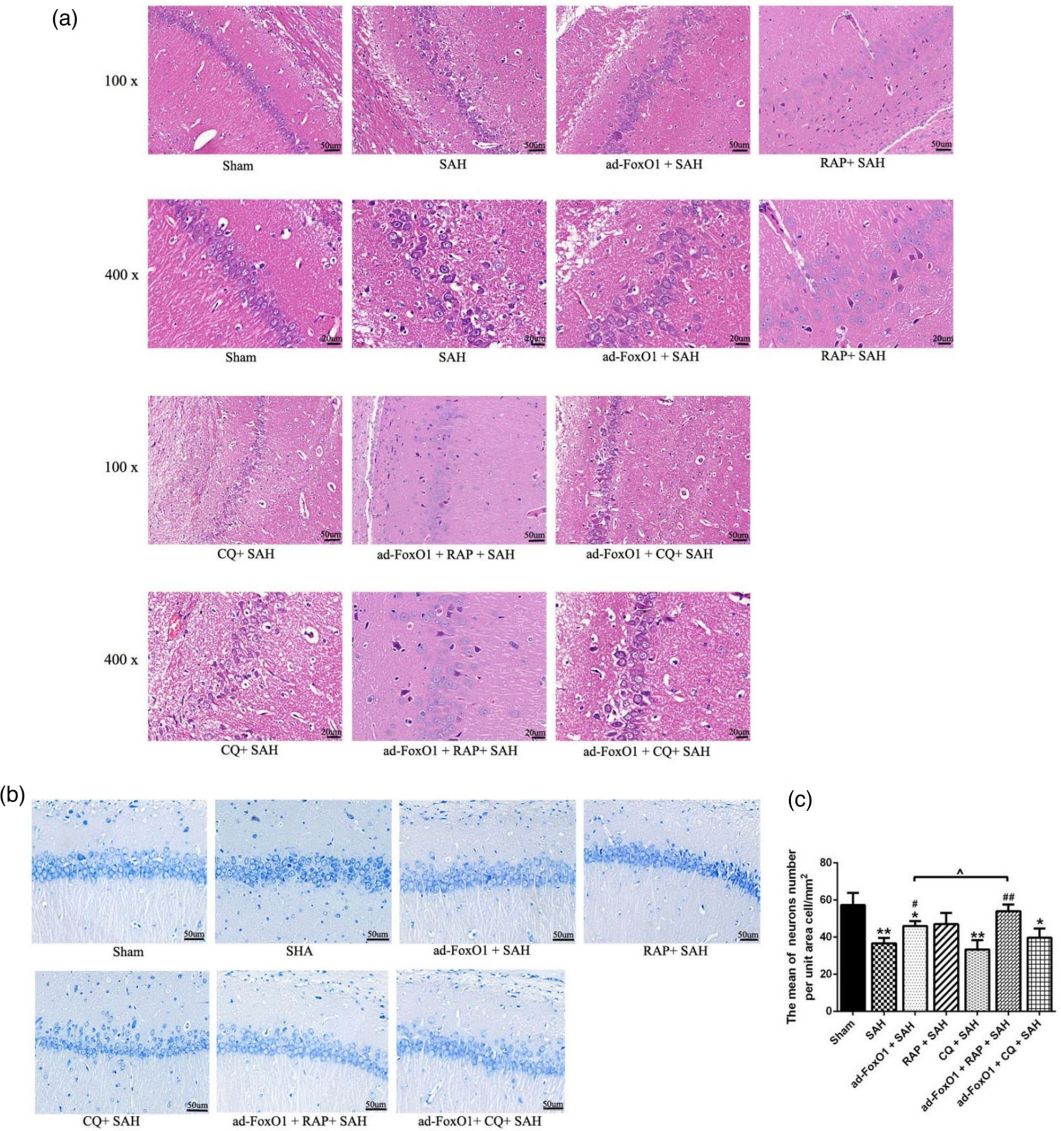
Ad‐FoxO1 improved pathomorphology and inhibited the neuronal apoptosis by involving in autophagy after SAH. (a) HE staining showed the pathomorphology of hippocampus after SAH. (b) Toluidine blue staining showed the qualitative histological changes of hippocampus after SAH (400×). (c) The neurons number was calculated in terms of per unit area cell/mm^2^. *n* = 6 in each group, *p < .05, **p < .01, vs. sham group. ^#^ *p* < .05, ^##^ *p* < .01, vs. SAH group. ˆ*p* < .05

HE staining results revealed that the sham‐operated rats had a tightly packed pyramidal layer of cells in the CA1 region of the hippocampus, and its neurons were in normal shape, accompanied by light‐colored nuclei, with no obvious degeneration or necrosis of neurons. In the SAH group, there were a large number of disarranged pyramidal cells in the hippocampal CA1 region, some of which were necrotic, and the necrotic cells were reduced in size and deepened in coloration, along with a large number of cell fragments. Besides, compared with SAH group, the ad‐FoxO1+SAH group, RAP+SAH group, and ad‐FoxO1+RAP+SAH group had varying increase of necrotic vertebral cells in CQ+SAH group and ad‐FoxO1+CQ+SAH group, with the greatest increase in CQ+SAH group. Moreover, compared with the ad‐FoxO1+SAH group, the pyramidal cells in the hippocampus CA1 region of ad‐FoxO1+RAP+SAH group were somewhat more orderly and with a less necrotic number of pyramidal cells, while ad‐FoxO1+CQ+SAH group with a more disordered arrangement and more necrotic number of pyramidal cells (Figure 6a). These results indicate that ad‐FoxO1 involving in autophagy regulation inhibited the degeneration and necrosis of pyramidal cells and decreased the number of cone cell in the hippocampal CA1 region.

The number of neurons in the hippocampus was further determined by toluidine blue staining to investigate the effects of ad‐FoxO1 involving in autophagy regulation on the number of neurons in the hippocampal CA1 region. It was confirmed that the number of neurons per unit area in SAH group were significantly lower than that in Sham group. Besides, the number of neurons in ad‐FoxO1+SAH group and ad‐FoxO1+RAP+SAH group were significantly higher than that in SAH group. In addition, compared with ad‐FoxO1+SAH group, the number of neurons was significantly increased in ad‐FoxO1+RAP+SAH group (Figure 6b,c).

### 3.7 Ad‐FoxO1 involved in autophagy regulation to reduce neuronal apoptosis during EBI following SAH

**FIGURE 7:**
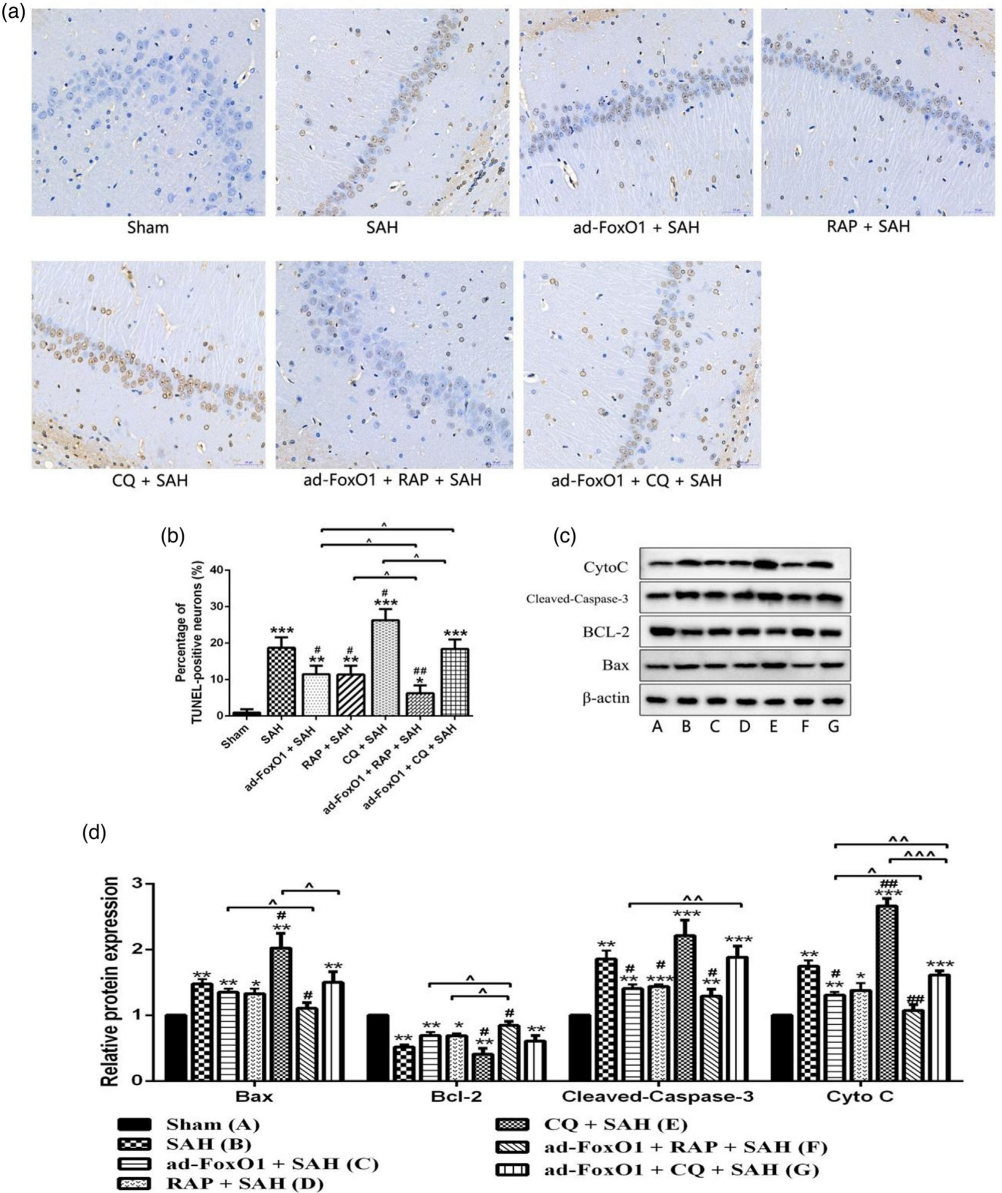
Ad‐FoxO1 involved in autophagy regulation to reduce neuronal apoptosis during EBI following SAH. (a) Representative images of hippocampus following TUNEL staining, nucleus of apoptotic cells were stained brown (400×). (b) Represents the change of neuronal apoptosis rate in each group. (c) Apoptosis‐related proteins were examined by western blotting assay. (d) The relative intensity of apoptosis‐related proteins was shown as a bar graph. *n* = 6 in each group, *p < .05, **p < .01, ***p < .001, vs. sham group. ^#^ *p* < .05, ^##^ *p* < .01, vs. SAH group. ˆ*p* < .05, ˆˆ*p* < .01, ˆˆˆ*p* < .001

Next, we examined whether ad‐FoxO1 involved in autophagy regulation to reduce the excessive neuronal apoptosis in the hippocampus CA1 region by TUNEL staining. Obviously, rats in CQ+SAH group showed higher percentage of TUNEL‐positive than SAH group, and rats in ad‐FoxO1 group and RAP+SAH group showed lower percentage of TUNEL‐positive than SAH group. Besides, compared to ad‐FoxO1+SAH group, there was a significantly reduction of neuronal apoptosis number in ad‐FoxO1+RAP+SAH group, and a significantly increase of neuronal apoptosis number in ad‐FoxO1+CQ+SAH group (Figure 7a,b).

Moreover, western blot results revealed that relative to sham group, expressions of Bax, Cleaved‐Caspase‐3, and CytoC in SAH group significantly increased, while expression of Bcl‐2 in SAH group significantly decrease. In terms of the Bax expression level, expression of Bax had a significantly higher level in CQ+SAH group and a significantly lower level in ad‐FoxO1+RAP+SAH group relative to SAH group. Besides, Bax expression level in ad‐FoxO1+RAP+SAH group was significantly lower than that in ad‐FoxO1+SAH group, and Bax expression level in ad‐FoxO1+CQ+SAH group was significantly lower than that in CQ+SAH group. In terms of the Bcl‐2 expression level, expression of Bcl‐2 had a significantly lower level in CQ+SAH group and a significantly higher level in ad‐FoxO1+RAP+SAH group relative to SAH group. Besides, Bcl‐2 expression level in ad‐FoxO1+RAP+SAH group was significantly higher than that in ad‐FoxO1+SAH group and in RAP+SAH group. In terms of the Cleaved‐caspase3 expression level, expressions of Cleaved‐caspase3 in ad‐FoxO1+SAH group, RAP+SAH group, and ad‐FoxO1+RAP+SAH group had significantly lower level relative to SAH group. And compared with ad‐FoxO1+SAH group, expression of Cleaved‐caspase3 in ad‐FoxO1+CQ+SAH group was significantly increased. In terms of the CytoC expression level, expressions of CytoC had significantly lower level in ad‐FoxO1+SAH group and ad‐FoxO1+RAP+SAH group and a significantly higher level in CQ+SAH group, relative to SAH group (Figure 7c,d).

### 3.8 Ad‐FoxO1 involved in autophagy regulation to attenuate the contents of inflammation‐related cytokines during EBI following SAH

**FIGURE 8:**
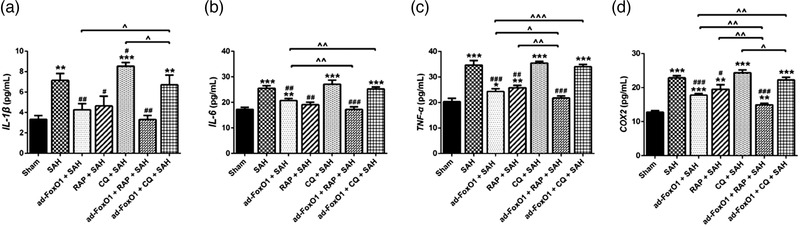
Ad‐FoxO1 involved in autophagy regulation to attenuate the inflammatory factor during EBI Following SAH. (a) The content of COX‐2 in each group. (b) The content of IL‐1β in each group. (c) The content of IL‐6 in each group. (d) The content of TNF‐α in each group. *n* = 6 in each group, *p < .05, **p < .01, and ***p < .001 vs. sham group. ^#^ *p* < .05, ^##^ *p* < .01 and ^###^ *p* < .001, vs. SAH group. ˆ*p* < .05, ˆˆ*p* < .01, ˆˆˆ*p* < .001

To investigate whether ad‐FoxO1 involved in autophagy regulation to attenuate the inflammatory response during EBI following SAH, inflammation‐related cytokines were further detected by ELISA. The results showed that, compared with the SAH group, the levels of inflammation‐related cytokines IL‐1β, IL‐6, TNF‐α, and COX2 decreased to different degrees in ad‐FoxO1+SAH group and RAP+SAH group, and increased to different degrees in the CQ+SAH group. Besides, relative to ad‐FoxO1+SAH group, the levels of COX2, IL‐1β, IL‐6, and TNF‐α had a significant downward trend in ad‐FoxO1+RAP+SAH group, and a significant upward trend in ad‐FoxO1+CQ+SAH group (Figure 8).

## 4 DISCUSSION

EBI after SAH refers to the occurrence of brain injury within 48 h after SAH (Wang Z. et al., 2011), and the mechanism of brain injury mainly starts from the pathophysiological changes in the brain caused by cerebral hemorrhage. EBI contains a range of molecular mechanisms, including the ischemic pathway, the apoptotic pathway, and the inflammatory pathway, which ultimately leads to a common outcome: cell death, BBB destruction, brain edema, and neuronal injury. Thus, improving EBI has become the main interference target to reduce EBI after SAH.

FoxO1 has been shown to play an important role in a variety of cells and tissues (Schiattarella & Altamirano, 2021). In recent years, this factor has been shown to be expressed in brain. In addition, the up‐regulation of FoxO1 expression is significant in improving neuroprotective function. For example, further investigation showed that the increase of FoxO1 is closely related with the inhibition of necroptosis in microglia cells after traumatic brain injury (Zhao P. et al., 2020). Besides, Calycosin‐7‐O‐β‐D‐glucoside (CG) alleviates OGD/R (oxygen‐glucose deprivation/reperfusion)‐induced brain injury by up‐regulating FoxO1 (Yan et al., 2019). Studies have shown that endogenous FoxO1 is a key regulator of autophagy in neurons (Vidal et al., 2012). FoxO1 has a unique function in inducing autophagy in various diseases, among which FoxO1 gene knockout can significantly inhibit autophagy (Friedman et al., 2021). Therefore, FoxO1 has a good clinical application prospect in neuroprotection. So we speculate that FoxO1 as a form of self‐protection reduces inflammation and thus protects the neural function of rats after SAH, and ad‐FoxO1 may have a protective effect on the body through autophagy regulatory mechanisms. Studies have shown that apoptosis after SAH is an important intracellular pathway leading to cell death and EBI, but whether FoxO1 protects the body through autophagy regulation is unclear. In the current study, we showed that FoxO1 protected against EBI via further promoting autophagy flux in a SAH model during EBI. Our data suggest that ad‐FoxO1 inhibits subsequent apoptotic insults, declines inflammatory response by promoting regulation of autophagy flow, and thus protects the function of neurons and improves brain damage. It has been reported that neuronal apoptosis is an important process leading to various pathologies of EBI (Sun et al., 2019). As a highly conserved biological phenomenon, the apoptosis degrades intracellular components and helps maintain cell homeostasis (Meng et al., 2019). In addition, autophagy activation has been shown to protect neurons from apoptosis after SAH. For example, recent studies have reported that autophagy enhancers reduce apoptosis, while autophagy inhibitors promote apoptosis after SAH induction (Guo D. et al., 2018; Li T. et al., 2015). Autophagy is an important protective mechanism against apoptosis in ischemic cell injury (Loos et al., 2011). Thus, the activation of autophagy would avoid apoptosis of neurons in EBI after SAH (Guo D. et al., 2018). Here, we provide a reasonable explanation for this phenomenon. Our findings indicate that the autophagy‐modulated apoptosis pathway is an important target for in EBI after SAH.

To obtain deeper insights into the mechanism of how FoxO1 protects the function of neurons and improves brain damage in SAH‐induced neurological deficits during EBI. We analyzed the effect of FoxO1 overexpress on nerve function recovery, oedema, BBB leakage, neuronal apoptosis, and autophagy activation in rats after SAH during EBI. In this study, either low or high dose of ad‐FoxO1 could significantly improve nerve function, reduce cerebral water content and reduce BBB destruction in rats, indicating that ad‐FoxO1 had a protective effect on brain injury in rats EBI after SAH, with an optimal neuroprotective effect highlighted by the high dose of ad‐FoxO1. Studies have shown that FoxO1 is upregulated after improvement of craniocerebral trauma (TBI). Stable knockdown of FoxO1 can increase the level of necroptosis after microglia damage (Zhao P. et al., 2020), and we speculate that it may be upregulated through autophagy for protective effects. One possible explanation is that in the hyperacute phase after SAH, injury‐related clearance systems, such as autophagy, are activated. FoxO1 is an autophagy regulatory gene, and its expression is increased to initiate autophagy flow patency, thus achieving a self‐regulatory mechanism to protect the body.

EBI after SAH involves a complicated pathophysiological process that involves a large number of pathways and factors. A variety of cell death mechanisms were linked to the pathogenesis of SAH, such as extrinsic and intrinsic apoptosis, (regulated) necrosis, and autophagy. A prominent feature of necrotic apoptosis is cell rupture, which can be identified by HE staining. HE staining results showed that the necrotic nerve cells in hippocampal tissues were mainly neurons. Toluidine blue staining was used to further identify the number of necrotic neurons. In addition, apoptosis of neuronal necrosis was observed by TUNEL staining. These results all suggest that ad‐FoxO1 has a certain correlation with neuronal necrotic apoptosis. In SAH rats, there are different degrees of apoptosis in hippocampus, BBB, and vascular system (Wei et al., 2020). It has been suggested that many experimental approaches protect against SAH‐induced brain injury by modulating caspas‐dependent neuronal death (Wang W. et al., 2019; Zhang Z. et al., 2018). Some scholars have found that apoptosis inhibitors can reduce the apoptosis of neurons to improve EBI after SAH (Gao et al., 2008). In this study, we found that ad‐FoxO1 involved in the apoptosis pathway of hippocampal tissues in the process of SAH rats during EBI. Bcl‐2 family proteins are the main regulatory factors in the process of apoptosis, mainly including apoptotic inhibitory proteins such as Bcl‐2 and pro‐apoptotic proteins such as Bax (Zhang Z. et al., 2018). In SAH, Bax expression was significantly increased, leading to an increase in Bax‐induced mitochondrial membrane permeability, followed by the release of Cytochrome C and Caspase9, which promoted the increased expression of activated Caspase‐3 and eventually led to neuronal apoptosis (Chen et al., 2014; Cho et al., 2015).

Our observation is therefore consistent with the general view set out above that the imbalance of Bcl2 and Bax protein expression after SAH was improved and apoptosis was reduced by treatment with ad‐FoxO1. Thus, inhibition of apoptosis in brain cells after SAH is considered to be an important link in the treatment strategy of SAH (Liu H. et al., 2016).

These apoptotic cells eventually undergo secondary necrosis, releasing pro‐inflammatory alarmins and affect the surrounding cells and environment. Other studies confirmed that the inflammatory response was also involved in the development of EBI (Zhang Z. Y. et al., 2017). Under the double influence of increased intracranial pressure and decreased cerebral blood flow, peripheral immune cells of rats after SAH infiltrated into brain tissues and released a large number of pro‐inflammatory factors, which not only damaged peripheral brain cells, but also damaged nerve cells and led to the occurrence of cerebral edema. The present study also observed that, rats after SAH could induce the expression of IL‐1β, IL‐6, TNF‐α, and COX2 in brain tissues, which was similar to previous studies (Zhao X. D. & Zhou, 2011). Besides, in this study, ad‐FoxO1 with low or high doses effectively inhibited inflammation. These results support our hypothesis that FoxO1 as a form of self‐protection reduces inflammation and thus protects the neural function of rats after SAH.

Autophagy, as a highly regulated way of intracellular component degradation, plays an important role in maintaining normal cell function, growth, and development. Endogenous FoxO1 is a key regulator of autophagy in neurons (Vidal et al., 2012). In addition, studies have shown that Beclin‐1 may be involved in the activation of Caspase‐3 (Küçükler et al., 2021). Bcl‐2 negatively regulates autophagy by inhibiting Bax and blocking Beclin‐1 (Lindqvist et al., 2014), indicating that there is mutual crosstalk between autophagy and apoptosis. Furthermore, autophagy and apoptosis may share a common molecular inducer that regulate the process of cell death (Nikoletopoulou et al., 2013). Recent studies have found that enhanced autophagy reduces levels of apoptosis and necrosis in models of hypoxic‐ischemic injury (Loos et al., 2011). In addition, the enhancement of autophagy also contributes to the reduction of neuronal apoptosis, the improvement of neurological function, and the reduction of EBI after SAH (Shao et al., 2016). In this study, we studied the effect of ad‐FoxO1 on autophagy in the hippocampus of SAH rats. Expression levels of LC3B II/LC3I and Beclin‐1 reflect the level of autophagy activation. P62 protein can be degraded by autophagy, so p62 can reflect the level of autophagy clearance. Results showed that ad‐FoxO1 promoted autophagy in rat hippocampal tissue, as evidenced by accumulation of LC3II/I and Beclin‐1 and degradation of p62, thus inhibiting the inflammatory response and apoptosis of rat hippocampal neurons, which is consistent with previous conclusion. These results provided the background for further studies on the effects of FoxO1 involving in autophagy on EBI in SAH rats.

Studies have shown that autophagy plays a protective role in EBI after SAH, since FoxO1 is a regulator of autophagy, and it plays a protective role in autophagy at 48 h before SAH. However, whether ad‐FoxO1 protects hippocampal nerve function in rats by involving in autophagy after SAH is not completely clear. Therefore, we designed another experiment, in which SAH rats treated with FoxO1 were intraperitoneally injected with RAP or CQ respectively. So far, most of the autophagy inducers discovered and widely used, such as RAP, a natural product, target the upstream of autophagy, which means that it can promote the occurrence of biological autophagosomes (Zhao H. et al., 2013), while CQ, as an autophagosome autophagy suppressor, can inhibit the occurrence of biological autophagosomes (Coryell et al., 2020). In our experiment, we found that both ad‐FoxO1 and RAP attenuated the injury of nerve function in rats during EBI after SAH, and this synergistic effect also further reduced cerebral edema and Evans blue extravasation, decreased hippocampus neuronal cell apoptosis, and declined inflammatory response. However, this was contrary to the results of CQ. In our experiment, though synergic effect between FoxO1 overexpression and RAP on autophagy and apoptosis is not as significant as their net effect on cerebral edema and nerve function. This indicates that FoxO1 and RAP may modulate neuronal functions via other mechanisms. For example, FOXO1 is also closely associated with oxidative stress. It has been reported that Calycosin‐7‐O‐β‐d‐glucoside (CG) protects neurons from apoptosis by regulating the SIRT1/FOXO1/PGC‐1 α pathway (Yan et al., 2019). In addition, RAP can also protect the autophagy mediated nerve by regulating PI3K/Akt1/mTOR/CREB signaling and antioxidant mechanisms (Singh et al., 2017). On the whole, FoxO1 may play an important role in maintaining homeostasis through autophagy. Therefore, our results indicate that FoxO1 has a protective effect on nerve function of EBI rats after SAH through regulating autophagy flux.

## 5 CONCLUSIONS

Our study extends the current understanding of autophagy in SAH‐induced EBI. We demonstrate that FoxO1 plays a protective role in EBI after SAH by involving in enhancing autophagy flux. This neuroprotective effect may be associated with autophagy mediated anti‐apoptotic mechanisms, which may provide new insights for SAH therapy.

## CONFLICT OF INTEREST

The authors declare that there is no conflict of interest regarding the publication of this article.

## AUTHOR CONTRIBUTIONS

Haitao Hao and Yahui Bai contributed to conception and design of the study. Haitao Hao, Yu Liu, and Junxin Liang contributed to acquisition, analysis, and interpretation of the data and writing the manuscript. Haitao Hao, Junxin Liang, and Shichao Guo discussed the results and implications and commented on the manuscript at all stages, as well as in the final approval of the version to be published.

### TRANSPARENT PEER REVIEW

The peer review history for this article is available at https://publons.com/publon/10.1002/brb3.2376

## Data Availability

The data used to support the findings of this study are available from the corresponding author upon request.

## References

[brb32376-bib-0001] Chen, J. , Wang, L. , Wu, C. , Hu, Q. , Gu, C. , Yan, F. , Li, J. , Yan, W. , & Chen, G. (2014). Melatonin‐enhanced autophagy protects against neural apoptosis via a mitochondrial pathway in early brain injury following a subarachnoid hemorrhage. Journal of Pineal Research, 56(1), 12–19. 10.1111/jpi.12086 24033352

[brb32376-bib-0002] Cho, H. , Jung, S. , Lee, G. , Cho, J. , & Choi, I. (2015). Neuroprotective effect of *Citrus unshiu* immature peel and nobiletin inhibiting hydrogen peroxide‐induced oxidative stress in HT22 murine hippocampal neuronal cells. Pharmacognosy Magazine, 11(Suppl 2), S284–S289. 10.4103/0973-1296.166047 26664016 PMC4653338

[brb32376-bib-0003] Coryell, P. R. , Goraya, S. K. , Griffin, K. A. , Redick, M. A. , Sisk, S. R. , & Purvis, J. E. (2020). Autophagy regulates the localization and degradation of p16(INK4a). Aging Cell, 19(7), e13171. 10.1111/acel.13171 32662244 PMC7370706

[brb32376-bib-0004] Friedman, B. , Corciulo, C. , Castro, C. M. , & Cronstein, B. N. (2021). Adenosine A2A receptor signaling promotes FoxO associated autophagy in chondrocytes. Scientific Reports, 11(1), 968. 10.1038/s41598-020-80244-x 33441836 PMC7806643

[brb32376-bib-0005] Gao, C. , Liu, X. , Liu, W. , Shi, H. , Zhao, Z. , Chen, H. , & Zhao, S. (2008). Anti‐apoptotic and neuroprotective effects of tetramethylpyrazine following subarachnoid hemorrhage in rats. Autonomic Neuroscience: Basic & Clinical, 141(1‐2), 22–30. 18558517 10.1016/j.autneu.2008.04.007

[brb32376-bib-0006] Gathier, C. S. , Dankbaar, J. W. , Mathieu, V. D. J. , Verweij, B. H. , Oldenbeuving, A. W. , Rinkel, G. J. E. , van den Bergh, W. M. , & Slooter, A. J. C. ; HIMALAIA Study Group . (2015). Effects of induced hypertension on cerebral perfusion in delayed cerebral ischemia after aneurysmal subarachnoid hemorrhage: A randomized clinical trial. Stroke; A Journal of Cerebral Circulation, 46(11), 3277–3281. 10.1161/STROKEAHA.115.010537 26443829

[brb32376-bib-0007] Guo, D. , Xie, J. , Zhao, J. , Huang, T. , Guo, X. , & Song, J. (2018). Resveratrol protects early brain injury after subarachnoid hemorrhage by activating autophagy and inhibiting apoptosis mediated by the Akt/mTOR pathway. Neuroreport, 29(5), 368–379. 10.1097/WNR.0000000000000975 29360689 PMC5851673

[brb32376-bib-0008] Guo, S. , Li, Y. , Wei, B. , Liu, W. , Li, R. , Cheng, W. , Zhang, X. , He, X. , Li, X. , & Duan, C. (2020). Tim‐3 deteriorates neuroinflammatory and neurocyte apoptosis after subarachnoid hemorrhage through the Nrf2/HMGB1 signaling pathway in rats. Aging, 12(21), 21161–21185. 10.18632/aging.103796 33168786 PMC7695377

[brb32376-bib-0009] Hariharan, N. , Maejima, Y. , Nakae, J. , Paik, J. , Depinho, R. A. , & Sadoshima, J. (2010). Deacetylation of FoxO by Sirt1 plays an essential role in mediating starvation‐induced autophagy in cardiac myocytes. Circulation Research, 107(12), 1470–1482. 10.1161/CIRCRESAHA.110.227371 20947830 PMC3011986

[brb32376-bib-0010] He, Y. , Cao, X. , Guo, P. , Li, X. , Shang, H. , Liu, J. , Xie, M. , Xu, Y. , & Liu, X. (2017). Quercetin induces autophagy via FOXO1‐dependent pathways and autophagy suppression enhances quercetin‐induced apoptosis in PASMCs in hypoxia. Free Radical Biology & Medicine, 103, 165–176. 27979659 10.1016/j.freeradbiomed.2016.12.016

[brb32376-bib-0011] Jellinger, K. A. , & Stadelmann, C. H. (2000). The enigma of cell death in neurodegenerative disorders. Journal of Neural Transmission Supplementum, (60), 21–36. 11205141 10.1007/978-3-7091-6301-6_2

[brb32376-bib-0012] Kim, T. W. (2021). Cinnamaldehyde induces autophagy‐mediated cell death through ER stress and epigenetic modification in gastric cancer cells. Acta Pharmacologica Sinica, 0, 1–12. 10.1038/s41401-021-00672-x PMC8888591 33980998

[brb32376-bib-0013] Küçükler, S. , Çomaklı, S. , Özdemir, S. , Çağlayan, C. , & Kandemir, F. M. (2021). Hesperidin protects against the chlorpyrifos‐induced chronic hepato‐renal toxicity in rats associated with oxidative stress, inflammation, apoptosis, autophagy, and up‐regulation of PARP‐1/VEGF. Environmental Toxicology, 36(8), 1600‐1617. 10.1002/tox.23156 33908150

[brb32376-bib-0014] Li, P. , Li, X. , Deng, P. , Wang, D. , Bai, X. , Li, Y. , Luo, C. , Belguise, K. , Wang, X. , Wei, X. , Xia, Z. , & Yi, B. (2020). Activation of adenosine A3 receptor reduces early brain injury by alleviating neuroinflammation after subarachnoid hemorrhage in elderly rats. Aging, 13(1), 694–713. 10.18632/aging.202178 33253120 PMC7835045

[brb32376-bib-0015] Li, T. , Sun, K.‐J. , Wang, H.‐D. , Zhou, M.‐L. , Ding, K.e , Lu, X.‐Y.u , Wei, W.u‐T. , Wang, C.‐X.i , & Zhou, X.‐M. (2015). Tert‐butylhydroquinone ameliorates early brain injury after experimental subarachnoid hemorrhage in mice by enhancing Nrf2‐independent autophagy. Neurochemical Research, 40(9), 1829–1838. 10.1007/s11064-015-1672-4 26260377

[brb32376-bib-0016] Li, X. , Liu, W. , Li, R. , Guo, S. , Fan, H. , Wei, B. , Zhang, X. , He, X. , & Duan, C. (2020). TSG‐6 attenuates oxidative stress‐induced early brain injury in subarachnoid hemorrhage partly by the HO‐1 and Nox2 Pathways. Journal of Stroke and Cerebrovascular Diseases: The Official Journal of National Stroke Association, 29(12), 104986. 10.1016/j.jstrokecerebrovasdis.2020.104986 32992175

[brb32376-bib-0017] Li, X. , Peng, J. , Pang, J. , Wu, Y. , Huang, X. , Li, Y. , Zhou, J. , Gu, L. , Sun, X. , Chen, L. , Vitek, M. P. , & Jiang, Y. (2018). Apolipoprotein e‐mimetic peptide COG1410 promotes autophagy by phosphorylating GSK‐3β in early brain injury following experimental subarachnoid hemorrhage. Frontiers in Neuroscience, 12, 127. 10.3389/fnins.2018.00127 29556174 PMC5844970

[brb32376-bib-0018] Lindqvist, L. M. , Heinlein, M. , Huang, D. C. S. , & Vaux, D. L. (2014). Prosurvival Bcl‐2 family members affect autophagy only indirectly, by inhibiting Bax and Bak. Proceedings of the National Academy of Sciences of the United States of America, 111(23), 8512–8517. 10.1073/pnas.1406425111 24912196 PMC4060681

[brb32376-bib-0019] Liu, H. , Yang, M. , Pan, L. , Liu, P. , & Ma, L. (2016). Hyperbaric oxygen intervention modulates early brain injury after experimental subarachnoid hemorrhage in rats: Possible involvement of TLR4/NF‐x03BA; B‐mediated signaling pathway. Cellular Physiology and Biochemistry: International Journal of Experimental Cellular Physiology, Biochemistry, and Pharmacology, 38(6), 2323–2336. 10.1159/000445586 27197977

[brb32376-bib-0020] Liu, L. , Zhang, P. , Zhang, Z. , Liang, Y. , Chen, H. , He, Z. , Sun, X. , Guo, Z. , & Deng, Y. (2021). 5‐Lipoxygenase inhibition reduces inflammation and neuronal apoptosis via AKT signaling after subarachnoid hemorrhage in rats. Aging, 13(8), 11752–11761. 10.18632/aging.202869 33878031 PMC8109136

[brb32376-bib-0021] Loos, B. , Genade, S. , Ellis, B. , Lochner, A. , & Engelbrecht, A.‐M. (2011). At the core of survival: Autophagy delays the onset of both apoptotic and necrotic cell death in a model of ischemic cell injury. Experimental Cell Research, 317(10), 1437–1453. 10.1016/j.yexcr.2011.03.011 21420401

[brb32376-bib-0022] Marbacher, S. (2016). Animal models for the study of subarachnoid hemorrhage: Are we moving towards increased standardization? Translational Stroke Research, 7(1), 1–2. 10.1007/s12975-015-0442-6 26754973

[brb32376-bib-0023] Meng, N. , Mu, X. , Lv, X. , Wang, L. , Li, N. , & Gong, Y. (2019). Autophagy represses fascaplysin‐induced apoptosis and angiogenesis inhibition via ROS and p8 in vascular endothelia cells. Biomedicine & Pharmacotherapy = Biomedecine & Pharmacotherapie, 114, 108866. 30999113 10.1016/j.biopha.2019.108866

[brb32376-bib-0024] Nikoletopoulou, V. , Markaki, M. , Palikaras, K. , & Tavernarakis, N. (2013). Crosstalk between apoptosis, necrosis and autophagy. Biochimica et Biophysica Acta, 1833(12), 3448–3459. 10.1016/j.bbamcr.2013.06.001 23770045

[brb32376-bib-0025] Nikoletopoulou, V. , Papandreou, M.‐E. , & Tavernarakis, N. (2015). Autophagy in the physiology and pathology of the central nervous system. Cell Death and Differentiation, 22(3), 398–407. 10.1038/cdd.2014.204 25526091 PMC4326580

[brb32376-bib-0026] Poillet‐Perez, L. , Despouy, G. , Delage‐Mourroux, R. , & Boyer‐Guittaut, M. (2015). Interplay between ROS and autophagy in cancer cells, from tumor initiation to cancer therapy. Redox Biology, 4, 184–192. 10.1016/j.redox.2014.12.003 25590798 PMC4803791

[brb32376-bib-0027] Schiattarella, G. G. , Altamirano, F. , Kim, S. Y. , Tong, D. , Ferdous, A. , Piristine, H. , Dasgupta, S. , Wang, X. , French, K. M. , Villalobos, E. , Spurgin, S. B. , Waldman, M. , Jiang, N. , May, H. I. , Hill, T. M. , Luo, Y. , Yoo, H. , Zaha, V. G. , Lavandero, S. , … Hill, J. A. (2021). Xbp1s‐FoxO1 axis governs lipid accumulation and contractile performance in heart failure with preserved ejection fraction. Nature Communications, 12(1), 1684. 10.1038/s41467-021-21931-9 PMC7966396 33727534

[brb32376-bib-0028] Shao, A. , Wang, Z. , Wu, H. , Dong, X. , Li, Y. , Tu, S. , Tang, J. , Zhao, M. , Zhang, J. , & Hong, Y. (2016). Enhancement of autophagy by histone deacetylase inhibitor trichostatin a ameliorates neuronal apoptosis after subarachnoid hemorrhage in rats. Molecular Neurobiology, 53(1), 18–27. 10.1007/s12035-014-8986-0 25399954

[brb32376-bib-0029] Shliapina, V. L. , Yurtaeva, S. V. , Rubtsova, M. P. , & Dontsova, O. A. (2021). At the crossroads: Mechanisms of apoptosis and autophagy in cell life and death. Acta Naturae, 13(2), 106–115. 10.32607/actanaturae.11208 PMC8327148 34377561

[brb32376-bib-0030] Singh, A. K. , Kashyap, M. P. , Tripathi, V. K. , Singh, S. , Garg, G. , & Rizvi, S. I. (2017). Neuroprotection through rapamycin‐induced activation of autophagy and PI3K/Akt1/mTOR/CREB signaling against amyloid‐β‐induced oxidative stress, synaptic/neurotransmission dysfunction, and neurodegeneration in adult rats. Molecular Neurobiology, 54(8), 5815–5828. 10.1007/s12035-016-0129-3 27660271

[brb32376-bib-0031] Sun, C. M. , Enkhjargal, B. , Reis, C. , Zhou, K.‐R. , Xie, Z.‐Y. , Wu, L.‐Y. , Zhang, T.‐Y. , Zhu, Q.‐Q. , Tang, J.‐P. , Jiang, X.‐D. , & Zhang, J. H. (2019). Osteopontin attenuates early brain injury through regulating autophagy‐apoptosis interaction after subarachnoid hemorrhage in rats. CNS Neuroscience & Therapeutics, 25(10), 1162–1172. 31436915 10.1111/cns.13199 PMC6776743

[brb32376-bib-0032] Tsuchiya, K. , Banks, A. S. , Liang, C.‐P. , Tabas, I. , Tall, A. R. , & Accili, D. (2011). Homozygosity for an allele encoding deacetylated FoxO1 protects macrophages from cholesterol‐induced inflammation without increasing apoptosis. Arteriosclerosis, Thrombosis, and Vascular Biology, 31(12), 2920–2928. 10.1161/ATVBAHA.110.219477 PMC3220790 21940942

[brb32376-bib-0033] Vidal, R. L. , Figueroa, A. , Court, F. A. , Thielen, P. , Molina, C. , Wirth, C. , Caballero, B. , Kiffin, R. , Segura‐Aguilar, J. , Cuervo, A. M. , Glimcher, L. H. , & Hetz, C. (2012). Targeting the UPR transcription factor XBP1 protects against Huntington's disease through the regulation of FoxO1 and autophagy. Human Molecular Genetics, 21(10), 2245–2262. 10.1093/hmg/dds040 22337954 PMC3335312

[brb32376-bib-0034] Wang, W. , Han, P. , Xie, R. , Yang, M. , Zhang, C. , Mi, Q. , Sun, B. , & Zhang, Z. (2019). TAT‐mGluR1 attenuation of neuronal apoptosis through prevention of MGluR1α truncation after experimental subarachnoid hemorrhage. ACS Chemical Neuroscience, 10(1), 746–756. 10.1021/acschemneuro.8b00531 30339347

[brb32376-bib-0035] Wang, Z. , Shi, X.‐Y. , Yin, J. , Zuo, G. , Zhang, J. , & Chen, G. (2012). Role of autophagy in early brain injury after experimental subarachnoid hemorrhage. Journal of Molecular Neuroscience: MN, 46(1), 192–202. 10.1007/s12031-011-9575-6 21728063

[brb32376-bib-0036] Wang, Z. , Zuo, G. , Shi, X. Y. , Zhang, J. , Fang, Q. , & Chen, G. (2011). Progesterone administration modulates cortical TLR4/NF‐κB signaling pathway after subarachnoid hemorrhage in male rats. Mediators of Inflammation, 2011, 848309. 21403869 10.1155/2011/848309 PMC3051156

[brb32376-bib-0037] Wei, C. , Guo, S. , Liu, W. , Jin, F.a , Wei, B. , Fan, H. , Su, H. , Liu, J. , Zhang, N. , Fang, D. , Li, G. , Shu, S. , Li, X. , He, X. , Zhang, X. , & Duan, C. (2020). Resolvin D1 ameliorates Inflammation‐mediated blood‐brain barrier disruption after subarachnoid hemorrhage in rats by modulating A20 and NLRP3 inflammasome. Frontiers in Pharmacology, 11, 610734. 10.3389/fphar.2020.610734 33732145 PMC7957930

[brb32376-bib-0038] Yan, X. , Yu, A. , Zheng, H. , Wang, S. , He, Y. , & Wang, L. (2019). Calycosin‐7‐O‐β‐D‐glucoside attenuates OGD/R‐induced damage by preventing oxidative stress and neuronal apoptosis via the SIRT1/FOXO1/PGC‐1α Pathway in HT22 cells. Neural Plasticity, 2019, 8798069. 31885537 10.1155/2019/8798069 PMC6915014

[brb32376-bib-0039] Yang, J. , & Wang, K. (2021). Vitamin D3 supplement attenuates blood‐brain barrier disruption and cognitive impairments in a rat model of traumatic brain injury. Neuromolecular Medicine, 0, 1–9. 10.1007/s12017-021-08649-z 33616826

[brb32376-bib-0040] Yang, Z. , & Klionsky, D. J. (2010). Mammalian autophagy: Core molecular machinery and signaling regulation. Current Opinion in Cell Biology, 22(2), 124–131. 10.1016/j.ceb.2009.11.014 20034776 PMC2854249

[brb32376-bib-0041] Zhang, L. , Wang, L.u , Xiao, H. , Gan, H. , Chen, H. , Zheng, S. , Jian, D. , Zhai, X. , Jiang, N. , Jing, Z. , & Liang, P. (2021). Tyrosine kinase Fyn promotes apoptosis after intracerebral hemorrhage in rats by activating Drp1 signaling. Journal of Molecular Medicine, 99(3), 359–371. 10.1007/s00109-020-02022-6 33409551

[brb32376-bib-0042] Zhang, X. S. , Wu, Q. , Wu, L. Y. , Ye, Z.‐N. , Jiang, T.‐W. , Li, W. , Zhuang, Z. , Zhou, M.‐L. , Zhang, X. , & Hang, C.‐H. (2016). Sirtuin 1 activation protects against early brain injury after experimental subarachnoid hemorrhage in rats. Cell Death & Disease, 7(10), e2416. 27735947 10.1038/cddis.2016.292 PMC5133967

[brb32376-bib-0043] Zhang, Z.‐Y. , Jiang, M. , Fang, J. , Yang, M.‐F. , Zhang, S. , Yin, Y.‐X. , Li, D.a‐W. , Mao, L.‐L. , Fu, X.‐Y. , Hou, Y.a‐J. , Fu, X.‐T. , Fan, C.‐D. , & Sun, B.‐L. (2017). Enhanced therapeutic potential of nano‐curcumin against subarachnoid hemorrhage‐induced blood‐brain barrier disruption through inhibition of inflammatory response and oxidative stress. Molecular Neurobiology, 54(1), 1–14. 10.1007/s12035-015-9635-y 26708209

[brb32376-bib-0044] Zhang, Z. , Liu, J. , Fan, C. , Mao, L. , Xie, R. , Wang, S. , Yang, M. , Yuan, H. , Yang, X. , Sun, J. , Wang, J. , Kong, J. , Huang, S. , & Sun, B. (2018). The GluN1/GluN2B NMDA receptor and metabotropic glutamate receptor 1 negative allosteric modulator has enhanced neuroprotection in a rat subarachnoid hemorrhage model. Experimental Neurology, 301(Pt A), 13–25. 10.1016/j.expneurol.2017.12.005 29258835

[brb32376-bib-0045] Zhao, H. , Ji, Z. , Tang, D. , Yan, C. , Zhao, W. , & Gao, C. (2013). Role of autophagy in early brain injury after subarachnoid hemorrhage in rats. Molecular Biology Reports, 40(2), 819–827. 10.1007/s11033-012-2120-z 23054025

[brb32376-bib-0046] Zhao, P. , Li, C. , Chen, B. , Sun, G. , Chao, H. , Tu, Y. , Bao, Z. , Fan, L. , Du, X. , & Ji, J. (2020). Up‐regulation of CHMP4B alleviates microglial necroptosis induced by traumatic brain injury. Journal of Cellular and Molecular Medicine, 24(15), 8466–8479. 32585748 10.1111/jcmm.15406 PMC7412706

[brb32376-bib-0047] Zhao, X.‐D. , & Zhou, Y.‐T. (2011). Effects of progesterone on intestinal inflammatory response and mucosa structure alterations following SAH in male rats. The Journal of Surgical Research, 171(1), e47‐e53. 10.1016/j.jss.2011.07.018 21924739

[brb32376-bib-0048] Zhao, Y. , Yang, J. , Liao, W. , Liu, X. , Zhang, H. , Wang, S. , Wang, D. , Feng, J. , Yu, L.i , & Zhu, W.‐G. (2010). Cytosolic FoxO1 is essential for the induction of autophagy and tumour suppressor activity. Nature Cell Biology, 12(7), 665–675. 10.1038/ncb2069 20543840

[brb32376-bib-0049] Zhou, K. , Enkhjargal, B. , Mo, J. , Zhang, T. , Zhu, Q. , Wu, P. , Reis, C. , Tang, J. , Zhang, J. H. , & Zhang, J. (2021). Dihydrolipoic acid enhances autophagy and alleviates neurological deficits after subarachnoid hemorrhage in rats. Experimental Neurology, 342, 113752. 10.1016/j.expneurol.2021.113752 33974879

[brb32376-bib-0050] Zhu, F. , Li, X. , Tang, X. , Jiang, J. , Han, Y.u , Li, Y. , Ma, C. , Liu, Z. , & He, Y. (2021). Neferine promotes the apoptosis of HNSCC through the accumulation of p62/SQSTM1 caused by autophagic flux inhibition. International Journal of Molecular Medicine, 48(1), Article 124. 10.3892/ijmm.2021.4957 33982791 PMC8128420

